# Hyalectanase Activities by the ADAMTS Metalloproteases

**DOI:** 10.3390/ijms22062988

**Published:** 2021-03-15

**Authors:** Tania Fontanil, Yamina Mohamedi, Jorge Espina-Casado, Álvaro J. Obaya, Teresa Cobo, Santiago Cal

**Affiliations:** 1 Departamento de Bioquímica y Biología Molecular, Universidad de Oviedo, 33006 Oviedo, Spain; taniuskina@gmail.com (T.F.); yamomu@hotmail.com (Y.M.); 2 Departamento de Investigación, Instituto Ordóñez, 33012 Oviedo, Spain; 3 Departamento de Química Física y Analítica, Universidad de Oviedo, 33006 Oviedo, Spain; jorge.espina@gmail.com; 4 Departamento de Biología Funcional, Área de Fisiología, Universidad de Oviedo, 33006 Oviedo, Spain; ajobaya@uniovi.es; 5 Instituto Universitario de Oncología, IUOPA, Universidad de Oviedo, 33006 Oviedo, Spain; 6 Departamento de Cirugía y Especialidades Médico-Quirúrgicas, Universidad de Oviedo, 33006 Oviedo, Spain; 7 Instituto Asturiano de Odontología, 33006 Oviedo, Spain

**Keywords:** aggrecan, versican, brevican, neurocan, hyalectan, lectican, proteoglycan, ADAMTS, extracellular matrix

## Abstract

The hyalectan family is composed of the proteoglycans aggrecan, versican, brevican and neurocan. Hyalectans, also known as lecticans, are components of the extracellular matrix of different tissues and play essential roles in key biological processes including skeletal development, and they are related to the correct maintenance of the vascular and central nervous system. For instance, hyalectans participate in the organization of structures such as perineural nets and in the regulation of neurite outgrowth or brain recovery following a traumatic injury. The ADAMTS (A Disintegrin and Metalloprotease domains, with thrombospondin motifs) family consists of 19 secreted metalloproteases. These enzymes also perform important roles in the structural organization and function of the extracellular matrix through interactions with other matrix components or as a consequence of their catalytic activity. In this regard, some of their preferred substrates are the hyalectans. In fact, ADAMTSs cleave hyalectans not only as a mechanism for clearance or turnover of proteoglycans but also to generate bioactive fragments which display specific functions. In this article we review some of the physiological and pathological effects derived from cleavages of hyalectans mediated by ADAMTSs.

## 1. Introduction

**Figure 1:**
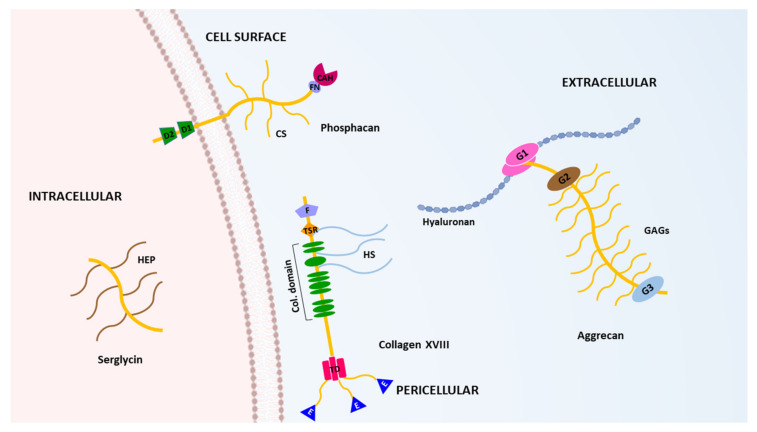
Schematic depiction of the structure of some representative proteoglycans. The complex domain organizations of an intracellular (serglycin), a cell surface-anchored (phosphacan), a pericellular (collagen type XVIII) and an extracellular (aggrecan) proteoglycan are shown. (HEP, heparin; D1 and D2, tyrosine phosphatase; FN, fibronectin-type III repeat; CAH, catabolic anhydrase domain; CS, chondroitin sulphate chains; F, frizzled domain; TSR, thrombospondin-like 1 domain; HS, heparan sulphate proteoglycans; Col. Domain, collagen domains; TD, trimerization domain; E, endostatin domain; G1, G2 and G3, globular domains; GAGs, glycosaminoglycans).

Proteoglycans (PGs) are crucial components of the extracellular matrix (ECM) of all tissues. Their complex structural architecture serves not only to provide a scaffold to support cells but also influences key biological events underlying processes such as cell differentiation, survival, proliferation and movement [1]. To perform those functions, PGs contain multiple glycosaminoglycans linked to a core protein containing a considerable number and variety of modular domains. Some examples of the modular architecture for representative PGs are shown in Figure 1. This intricate structure contributes to form molecular bridges between cells and the ECM, and also serves to bind cytokines and different growth factors, which allows the participation of PGs in the regulation of cell behavior in normal and pathological conditions. Moreover, PGs composition is different among tissues, indicating their involvement in particular roles within each specific tissue. Functional relevance of PGs in the ECM assembly is also evident when different pathological processes have been related with alterations affecting their physiological role [1,2]. For instance, different studies point out that the interaction of PGs with molecular partners of the ECM is essential for the correct maintenance of neuronal functions and the development of the nervous system [3,4,5,6]. Consequently, PGs have been associated with the development of different neurodegenerative disorders including those resulting in cognitive impairment as occurs in Alzheimer’s disease [7]. The influence of PGs has also been widely documented in other pathologies [2]. In cancer, PGs contribute to the generation of a profoundly remodeled ECM during tumorigenesis [8,9,10], but they can also act as pleiotropic modulators of different signaling pathways [11]. Cancer-associated fibroblasts are mainly responsible for those changes. These fibroblasts, in cooperation with cancer cells, support inflammation and can also secrete ECM components and ECM-remodeling enzymes which will ultimately promote tumor progression [12]. In fact, most of the modifications occurring in the ECM during tumorigenesis increase its stiffness and induce immunosuppressive responses [13]. However, different antitumor effects have also been associated to the ECM in cancer, in which PGs have been involved [13,14].

PGs are also main components of the ECM in cartilage where they are necessary to preserve its biomechanical properties [15,16,17]. Thus, PGs are essential to absorb compressive loads but also to modulate signaling pathways or to accumulate regulatory factors in the connective tissue. Consequently, an unregulated degradation of PGs promotes inflammation thereby contributing to the unwanted effects of osteoarthritis. PGs have also been related to the development of fibrosis through their relationship with the formation of collagen networks. In fact, collagen displays a highly disorganized structure in fibrosis disease, exhibiting a high density of cross-linking [18]. Due to that, PGs affect compaction of collagen [19]; they can also act as modulators of fibrosis [20,21].

Relationship between PGs and ECM diseases is also evident when mutations in 11 of 35 genes coding for PGs have been involved in the development of genetic disorders [22]. Some well-characterized examples include mutations in the genes encoding versican, which provokes erosive vitreoretinopathy and Wagner disease [23]; biglycan, which causes Meester-Loeys syndrome characterized by skeletal anomalies or aortic aneurysm [24]; perlecan, related to the Schwartz-Jampel syndrome characterized by skeletal dysplasia and myotonia [25]; or nyctalopin, involved in a type of night blindness [26].

These are just a few examples that illustrate the importance of PGs in the biology of the ECM. A deep knowledge of the functions of PGs would shed light about the molecular mechanisms associated to abnormal ECM remodeling [1]. Versican, aggrecan, neurocan and brevican are proteoglycans which form the group of the hyalectans. Given the importance of the hyalectans in the homeostasis of the ECM from different tissues, this review is focused on the effects related to their proteolysis by the ADAMTSs (A Disintegrin and metalloprotease domains, with thrombospondin motifs) metalloproteases.

## 2. The ADAMTSs Metalloproteases

**Figure 2:**
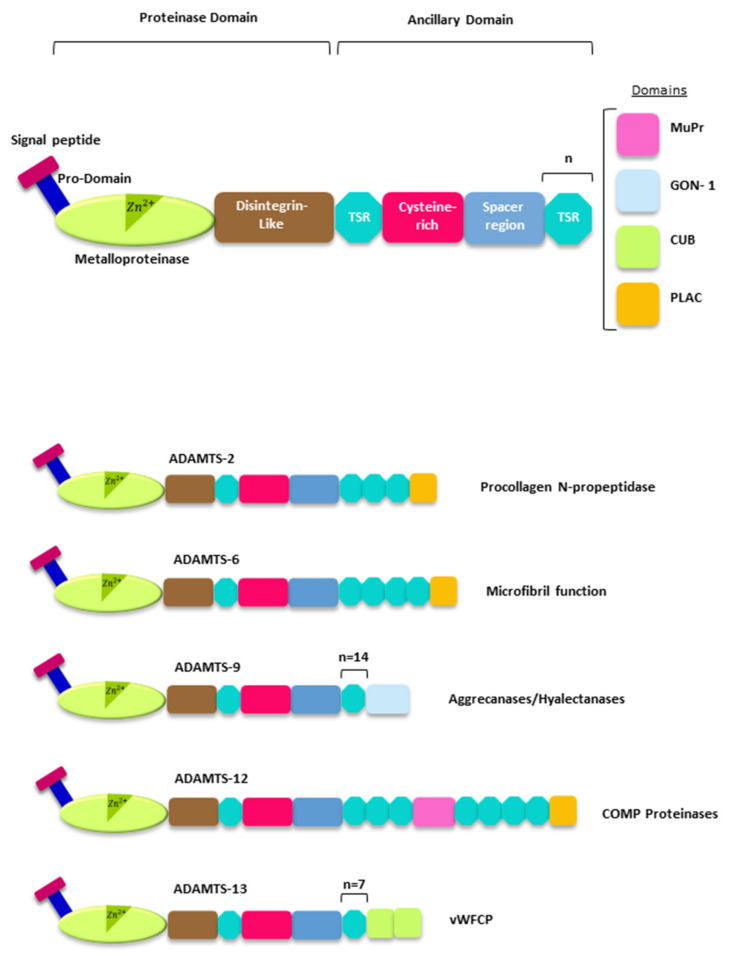
The ADAMTS family of metalloproteases. Top, schematic representation of the structure of the ADAMTS metalloproteases. TSR, thrombospondin type I motif. MuPr, mucin/proteoglycan domain. GON-1, CUB and PLAC domains (“*n*”, number of repetitions of the indicated domain). Bottom, representative members of the ADAMTS family from each of the functional subgroups: procollagen *N*-propeptidase, aggrecanases/hyalectanases, COMP proteinases, vWFCP and ADAMTSs (A Disintegrin and Metalloprotease domains, with thrombospondin motifs) related to microfibril function. For detailed information about the molecular architecture of the ADAMTS metalloproteases see reference [28].

The ADAMTS family comprises 19 human secreted metalloproteases. Structurally, all ADAMTSs share a complex molecular architecture and two regions are distinguishable: a proteinase domain and an ancillary domain [27,28]. In turn, the catalytic domain includes a signal, propeptide, a metalloproteinase (which contains the active site of the enzyme), and a disintegrin-like domain. This catalytic domain expands the amino-terminal region of the enzyme. The ancillary domain shows the greatest structural variability among all ADAMTSs, and it contains a central thrombospondin type 1 motif (TSR), a cystein-rich domain, a spacer domain and a variable number of TSR domains. Some particular ADAMTSs also displayed more specialized domains at the carboxy-terminal region (Figure 2). The ancillary domain is essential to interact with other ECM components and to control the catalytic activity of the enzyme.

Phylogenetic analysis allows assembling the 19 human ADAMTSs in eight different subgroups which makes possible an overview of the functions performed by these enzymes [28]. One of those subgroups includes ADAMTS-1, 4, 5, 8, 9, 15 and 20, metalloproteases, which are able to cleave PGs, including the hyalectans [27]. Another subgroup includes ADAMTS-2, 3 and 14, which are involved in the process of maturation of collagen [29]. ADAMTS-7 and ADAMTS-12 can cleave cartilage oligomeric matrix protein (COMP) [30,31]. The only member of another subgroup, ADAMTS-13, also known as von Willebrand factor-cleaving proteases (vWFCP), is involved in the processing of the von-Willebrand factor and is related to blood coagulation anomalies [32]. ADAMTS-6 and ADAMTS-10; ADAMTS-17 and ADAMTS-19; and ADAMTS-16 and ADAMTS-18 conform the other subgroups attending to their similar domain organization [28]. Members of these three last subgroups have often been classified as orphan ADAMTSs due to the difficulty of identifying their natural subtrates. However, recent studies have made it possible to connect some of these ADAMTSs with fibrillin function [33]. This is the case of ADAMTS-10, which may act as a fibrillinase, thus contributing to the microfibril formation process [34]. ADAMTS-6, the closest partner of ADAMTS-10, is able to cleave the microfibril-associated LTBP1 and also syndecan 4, and disrupts the heparan suphate-rich interfaces involved in microfibril deposition [35]. Characterization of an ADAMTS-17 variant causing Weill–Marchesani syndrome 4 has allowed suggesting that fibrillin-1 and fibronectin could be potential substrates for this metalloprotease [36].

The relationship between mutations in the gene coding for ADAMTS-17 and for ADAMTS-10 with Weill–Marchesani syndrome had been previously described [37]. This syndrome is characterized by anomalies of the lens in the eye, short stature and joint stiffness. Knobloch syndrome is caused by mutations in the gene coding for ADAMTS-18, which is characterized by severe myopia, vitreo-retinal degeneraton and skull abnormatilies [38,39]. Mutations in the gene coding for ADAMTS-16 have been associated with aberrant renal development or male infertility in mouse [40,41,42].

Taking together, these data highlight the importance of the ADAMTSs in relevant biological functions and their relationship to different inherited and acquired human disorders [43], a deeper knowledge about the participation of these metalloproteases in ECM remodeling through the cleavage of hyalectans is an important task to shed light on their specific roles in both normal and pathological conditions.

## 3. The Hyalectans

Versican, aggrecan, neurocan and brevican are PGs which form the group of hyalectans, also known as lecticans, and participate in the organization of the ECM of different tissues. For instance, hyalectans and their interacting partners contribute to form a three-dimensional network to control neurite outgrowth in the central nervous system (CNS) [44,45]. Structurally, hyalectans share a common hallmark consisting in a central region containing attachment sites for glycosaminoglycans. Two globular domains flank this central region, G1, at the NH_2_-terminal end; and G3, at the COOH-terminal end. An additional G2 domain, which is close to the G1 domain, can be identified in aggrecan. The G1 domain is linked to hyaluronan and forms interactions with other proteins. G3 contains epidermal growth factors repeats and a C-type lectin domain. The G3 domain also mediates the binding to other ECM components such as tenascin-R and tenascin-C. Those structural features led to name these PGs hyalectans (hyaluronan and lectin) [46].

Core protein of hyalectans ranges between about 145 kDa and more than 300 kDa [47]. However, it is common to identify smaller fragments since hyalectans undergo frequent cleavage by different proteolytic enzymes [44]. Matrix metalloproteinases (MMPs) and ADAMTSs, which can be recognized as proteoglycanases [47], are responsible for those cleavages but show remarkable differences. An illustrative example is that both MMPs and ADAMTSs can cleave aggrecan in the region located between the G1 and G2 domains, called the interglobular domain (IGD), but at different sites [48]. Thus, MMPs mainly cleave at the N360-F361 bond, whilst ADAMTSs cleave IGD at the E392-A393 bond. This difference has allowed generating antibodies to recognize the new terminal sequences created following cleavage of aggrecan (neoepitopes), and therefore to attribute the proteolysis to the appropriate (PLEASE, replace by “appropriate”) family of metalloproteases [47]. But aggrecan can be cleaved at other sites and this is the reason why smaller fragments can be detected depending on the antibody employed to detect aggrecanolytic activity.

Similarly to what happens with aggrecan can occur with the rest of hyalectans, affecting their functional activity. In this regard, cleavage of hyalectans by ADAMTSs has been related to different pathological situations, of which the main ones are reviewed below.

## 4. Hyalectans Cleavage by ADAMTSs

In 1997, Kuno et al. [49] performed the screening of genes which were selectively expressed in colon cachexia cell lines. Their work led to the identification of the first member of the ADAMTS family, ADAMTS-1, which was associated with inflammatory processes. More than twenty years later, different substrates for most of the 19 ADAMTSs metalloproteases have been identified, which has allowed associating ADAMTSs to different physiological processes. Among these substrates, the four hyalectans have been a fundamental research topic since these PGs are important modulators of the ECM, performing essential roles in physiological and pathological situations.

### 4.1. Cleavage of Aggrecan by ADAMTSs

**Figure 3:**
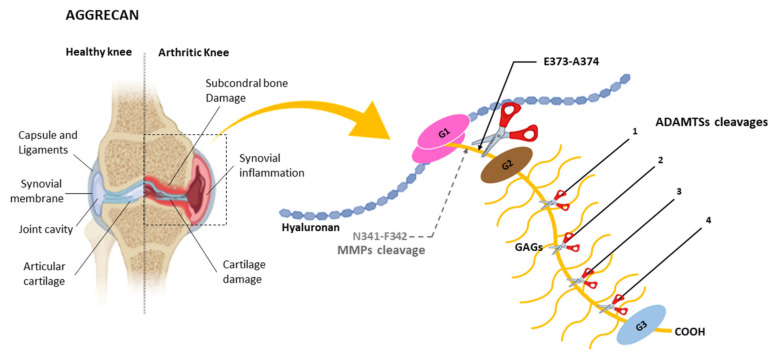
Schematic representation of an arthritic knee and aggrecan. G1, G2 and G3 indicate globular domains. Cleavage at the E373-A374 bond by the ADAMTSs in the interglobular domain (IGD) between G1 and G2 domains is indicated. This position is related to the development of juvenile joint diseases, causing inflammation in parts of the body like the knee. GAGs indicates glycosaminoglycans. (This position corresponds to the E392-A374 bond in reference [48]). Relative positions of additional cleavages sites (1,2,3,4) within the chondroitin sulphate-rich region are also indicated. These positions correspond to the E1279-G1280, E1467-L1468, E1572-A1573 and E1672-L1673 bonds identified in mouse cartilage aggrecan as shown in reference [72]. Main cleavage site by MMPs (N341-F342) in the IGD domain is also indicated. (This position corresponds to the N360-F361 bond in reference [48]).

Aggrecan is a major component of articular cartilage, and its uncontrolled degradation can cause degenerative joint diseases. In 1992, Sandy et al. [50] published a seminal article reporting the identification of aggrecan fragments from patients with a knee injury. Following purification of those fragments, the NH_2_-terminal sequencing revealed the sequence NH_2_-ARGSV (Figure 3), compatible with a cleavage at the E373-A374 bond within the IGD domain, which corresponds with the E392-A393 bond attending to the most recent UniProt nomenclature (see ID:P16112 at uniprot.org; accessed on 16 December 2020).

The identification of the “aggrecanase” responsible for the cleavage at this site, different from the site where the MMPs cleave aggrecan [48], could open new therapeutic opportunities for the treatment of juvenile joint diseases. In 1999, Tortorella et al. [51] and Abbaszade et al. [52] could assign that aggrecanase activity to two members of the ADAMTS family, ADAMTS-4 (aggrecanase-1) and ADAMTS-5 (aggrecanase-2) (ADAMTS-5 was initially called ADAMTS-11). Since then, ADAMTS-4 and ADAMTS-5 were considered appropriate targets in therapies to fight osteoarthritic diseases [53]. Importance of the cleavage at the E373-A374 bond in the development of cartilage pathology was validated by the use of genetically modified mice. Generation of knock-in mice containing a mutation that impedes cleavage at that bond confers protection against the development of cartilage lesions [54]. Moreover, resistance to the cleavage at this aggrecanase site induces higher nanodymaic stiffness magnitude and lower hydraulic permeability in mice as compared to wild-type controls, indicating protection from joint-overuse in distal femur cartilage [55]. It is also noteworthy than ADAMTSs other than ADAMTS-4 and ADAMTS-5 are able to cleave aggrecan at the E373-A374 bond [50,56]. Advances in the knowledge of ADAMTS biology and their relationship with the arthritic diseases have made it possible to develop therapeutic strategies to block the unwanted action of the ADAMTSs. Especially in regard to ADAMTS-5, which is recognized as the main aggrecanase and whose recent 20th anniversary was celebrated with a review by Santamaria, S. [57]. In relation to those strategies, inhibitors containing a zinc-binding group could block aggrecanase activities. However, these products show poor selectivity and bioavailability after oral administration [58]. A different strategy consists of blocking specific amino acids within the ancillary domain that participate in substrate recognition. Recently, two regions essential to cleave not only aggrecan but also versican have been identified within the spacer domain of ADAMTS-5 [59]. This finding opens the possibility to specifically block ADAMTS-5 activity by small molecules or preferably, by antibodies [60]. In fact, employment of antibodies against ADAMTS-5, some of which have entered a phase I clinical trial, emerges as a promising therapeutic option against arthritic diseases [61,62,63].

The E373-A374 bond at the IGD domain is not the only site that can be cleaved by aggrecanases, as could be inferred by the characterization of animal models lacking those activities. Mice lacking an active ADAMTS-5 show an important protection towards aggrecan proteolysis at the E373-A374 bond [64,65]. Also, mice lacking both ADAMTS-4 and ADAMTS-5 activities are protected from developing pathologies related with cleavage at the E373-A374 bond [66,67]. Moreover, these mice show normal skeletal development and similar effects upon aggrecan degradation as mice lacking only ADAMTS-5. However, aggrecan cleavage is still happening in these mice, but in sites other than the E373-A374 bond [68]. Consistent with those observations, four additional sites have been identified within the large chondroitin sulphate-region located between the G2 and G3 domains [69] (Figure 3). All these sites contain a glutamic acid in the upstream position [70]. The downstream position is more variable and alanine, leucine or glycine residues can be found there. Moreover, ADAMTSs other than ADAMTS-4 and ADAMTS-5 can also display aggrecanase activity. For instance, ADAMTS-1 can cleave aggrecan at four different sites in human and rat aggrecan [71]. More recently, ADAMTS-9 has been identified as a metalloprotease containing aggrecanase activity in mice lacking ADAMTS-4 and ADAMTS-5 activities [72]. It is also noteworthy that ADAMTS-9 expression can be induced with retinoic acid but not with interleukin-1α, contrary to what happens with ADAMTS-4 and ADAMTS-5 [67]. Moreover, ADAMTS-9 preferentially cleaves aggrecan at the E1279-G1280 and E1467-G1468 bonds within the chondroitin sulphate-region rather than the E373-A374 bond at the IGD domain. It has been proposed that ADAMTS-9 activity on aggrecan could be related to the normal turnover of the hyalectan in cartilage and not with a pathological condition as occurs with the aggrecanases ADAMTS-4 and ADAMTS-5 [72].

The relationship between ADAMTSs and aggrecan was explored not only in pathologies like degenerative joint diseases but also in vascular alterations. In fact, aggrecan plays important roles in the developing of the cardiovascular system and it has been related to disorders such as atherosclerosis, vascular re-stenosis or aortic aneurysms [73]. Suna et al. [74] reported that an upregulation of aggrecan and presence of aggrecan fragments take place following stent implantation in stretched coronary arteries in pigs. These effects were accompanied by changes in the levels of ADAMTSs gene expression since an increase of ADAMTS-4 and reductions of ADAMTS-1 and ADAMTS-5 levels were detected [75]. In mice lacking ADAMTS-5 activity, accumulation of aggrecan together with dilation of thoracic aorta was also found, suggesting the involvement of this metalloprotease in vascular remodeling. A later study confirmed that mice lacking ADAMTS-5 show aortic anomalies, which are due to an accumulation of aggrecan near the intimal layer and within the medial layer in the aortic wall [76]. Fragments derived from aggrecan could also be detected, but corresponding to cleavages at the sites in the chondroitin sulphate-region. Consequently, cleavage at the E373-A374 bond by ADAMTS-5 in the IGD domain of aggrecan could be essential for normal development of the aortic wall.

Increased levels of ADAMTSs and aggrecan have been associated with other pathological processes. Thus, it could be determined that ADAMTS-1 and aggrecan levels are increased in serum of adolescents and younger-aged females with polycystic ovary [77]. In ovarian cancer, ADAMTS-1 and ADAMTS-5 levels have also been found increased, and aggrecan levels are raised in malignant subtypes when compared to benign ovarian cancer [78]. These studies have been focused on the predictive potential of the detection of ADAMTSs and some of their substrates in these pathologies. However, presence and type of aggrecan fragments remain to be analyzed. Moreover, aggrecan is also detected in brain associated to perineural nets [79], and ADAMTSs are found to perform essential functions in the CNS [80]. Characterization of aggrecan cleavage by ADAMTSs in the brain could help to shed light on the mechanisms involved in brain plasticity and disease. Further studies aimed to identify and validate aggrecan fragments would help to better understand the specific role of ADAMTSs in those processes.

### 4.2. Cleavage of Versican by ADAMTSs

Versican is a major component of the ECM of adult and embryo tissues and is crucial for a correct tissue morphogenesis and organogenesis [81], and its expression has been detected in both a transient and stable manner. Thus, in the late 1990s, versican expression was detected in different parts of the developing mouse [82] and chick [83] hearts, that would indicate that this hyalectan displays important roles in cardiogenesis. At present, a large number of studies have corroborated the functional relevance of versican not only in normal heart function, but also in limb development or neural cell crest migration, among others [81,84].

**Figure 4:**
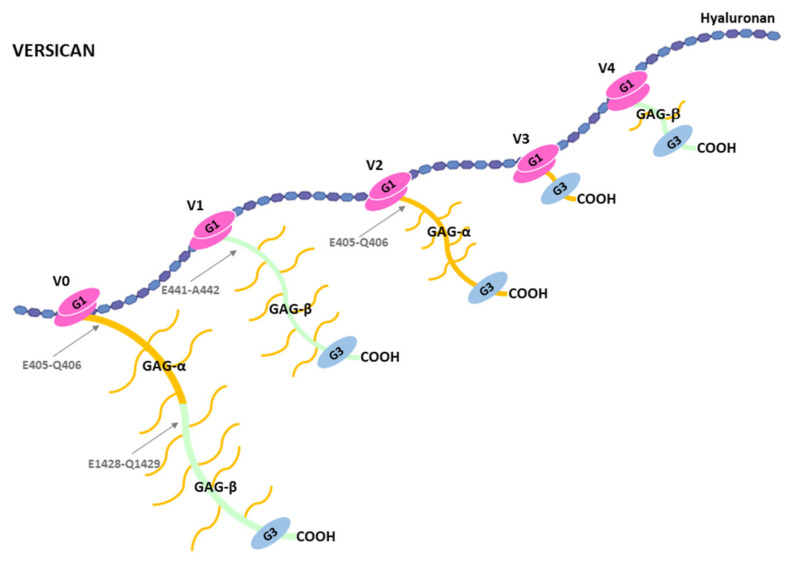
Schematic representation of the different isoforms of versican V0, V1, V2, V3 and V4. GAG-α and GAG-β are the two different glycosaminoglycans binding sites between the G1 and G3 globular domains. Arrows indicate some cleavage sites in versican. E405-Q406 is cleavage site in the GAG-α both in versican V0 and V2. E1428-Q1429 in versican V0 is the equivalent to E441-A442 in human versican V1 (see reference [20]).

Structurally, versican occurs in five different isoforms, V0, V1, V2, V3 and V4, due to different splicings events [84]. V0 is the largest isoform and it contains two globular domains, G1 and G3 at the NH_2_-terminal and COOH-terminal ends respectively (Figure 4). A central area is located between these two globular domains and it contains two regions with attachment sites for GAGs, GAG-α and GAG-β. The V1 isoform lacks GAGα, V2 lacks GAG-β, V3 lacks both GAG-α and GAG-β; that is why it does not contain anchored GAGs, and V4 lacks GAG-α and contains a shorter GAG-β, corresponding to its NH_2_-terminal part. Variations in the expression of these isoforms are found among different tissues and stages of development in normal physiological conditions. For instance, versican V0 and V1 are found highly expressed during embryonic development, and V2 is expressed after birth and is mainly located in the brain [81,85,86,87]. Versican V3 expression is scarcely found at the protein level, but its expression is upregulated upon induction with specific growth factors or cytokines in endothelial cells [88]. Moreover, versican V3 exogenously expressed in rat arterial smooth muscle cells increases adhesion and inhibition of migration and proliferation, which is contrary to the effects induced by other variants of versican [89]. V4 isoform is found in breast cancer tissues, which could offer potential therapeutic options to target versican in breast tumors [90].

In addition to the variability of versican forms due to the alternative splicing events, versican undergoes proteolytic cleavages by different proteases including MMPs and ADAMTSs [81]. In reference to the latter, ADAMTS-1, 4, 5, 9, 15 and 20 are able to cleave versican, and similarly to what happens with the name aggrecanase, the term “versicanase” has been coined to describe that property. Both, versican and ADAMTSs have been related to different functions in the cardiovascular system and some consequences of the versican proteolysis have been revealed [91]. For instance, ADAMTS-1 and ADAMTS-4 versicanase activity was evaluated in a study to characterize the presence of a 70-kDa versican fragment in mature human aorta [92]. These proteases were able to cleave both recombinant and native versican at the E441-A442 bond. This cleavage was coincident with that of the aorta versican V1 fragment, which could be identified through the use of antiserum to the COOH-terminal neoepitope generated upon cleavage, DPEAAE-COOH. The 70-kDa versican V1 fragment derived from ADAMTS-1 proteolysis has been associated with endocardial cells undergoing epithelium-mesenchymal transition and to the newly formed mesenchymal cells [93]. Also, ADAMTS-1 is fundamental for myocardial morphogenesis [94], and the generation of the 70-kDa versican V1 product seems to play an essential role in the formation of embryonic myocardium. Cleavage of versican by ADAMTS-1 has also been linked to pathological processes in the cardivascular system. In fact, ADAMTS-1 has been associated with the promotion of atherogenesis and to an increase of migration of primary aortic vascular smooth muscle cells [95]. ADAMTS-1 has also been related to the early phase of acute myocardial infarction [96]. Nevertheless, in this case it is suggested that unknown substrates, but not versican, can be cleaved by the metalloprotease. In relation to ADAMTS-4, its versicanase activity has also been examined in diet induced atherosclerotic plaques [97]. Employment of mice lacking ADAMTS-4 showed a reduced versican degradation, which contributes to increase plaque stability. ADAMTS-15 and ADAMTS-5 ability to cleave versican could also be important for the cardiovascular system function. Thus, versican proteolysis by ADAMTS-15 at the E441-A442 bond has been described and suggested to be relevant for embryonic heart as well as musculoskeletal development [98]. In relation to ADAMTS-5, mice lacking this metalloprotease exhibit ascending aortic anomalies [76,99], and show altered versican cleavage related to myxomatous valve disease [100].

ADAMTSs-mediated versicanase activity performs essential functions not only in the cardiovascular system. Generation of knock-in mice with a versican resistant to the cleavage by ADAMTSs have revealed new functional links between ADAMTSs and versican. These mice exhibit syndactyly and an increased deposition of type I and III collagens and hyaluronan, which is related to an accelerated wound-healing process [101]. These finding would indicate that cleavage sites in versican determines its turnover in the ECM. In relation to the bioactive fragments, the 70-kDa fragment derived from the cleavage of versican V1 by ADAMTSs strongly interacts with hyaluronan-rich cumulus ECM in porcine ovary [102]. Moreover, a second fragment showing the same neoepitope and of 65-kDa has been identified associated to mural granulosa cells in the same study. These results could be relevant to understand different aspects related to female infertility [102]. Versicanase activity is also related to the closure of mouse palate [103]. Development of animal models to investigate functional relevance of ADAMTS-9 and ADAMTS-20 as well as of versican has allowed determining that a defective versican cleavage by these proteases is related with a cleft palate phenotype. A cooperative mechanism involving versican cleavage in vascular endothelium by ADAMTS-9 and in palate mesenchyme by ADAMTS-20 would be necessary for a correct palate closure. Animal models have also made it possible to show that proteolytic action of ADAMTSs on versican regulate interdigital web regression [104]. ADAMTS-5, ADAMTS-9 and ADAMTS-20 seem to play a crucial role in that process as the combination of null alleles of the genes coding for these proteases result in soft-tissue syndactyly. Nevertheless, employment of a recombinant 70-kDa versican corresponding to the DPEAAE-COOH fragment generated by ADAMTSs cleavage on versican V1 induced apoptosis in the webs in mice lacking ADAMTS-5, the ADAMTS shows the strongest versicanase activity [59]. Consequently, versican cleavage by ADAMTSs and in particular by ADAMTS-5 could induce apoptosis of interdigital webs.

Fragments derived from versican proteolysis have also been identified in other pathological contexts. Thus, induction of liver fibrosis in mice using treatment with carbon tetrachloride during 4 weeks led to an increase in both versican and versicanase levels [20]. These levels decreased once the treatment was stopped, but an increase of versicanase activity was again detected after 28 days, particularly that of ADAMTS-8. Presence of the DPEAAE-COOH neoepitope was analyzed, with the finding that the fragment derived from V1 versican proteolysis was detected during the experimental process, but showed a decrease after 28 days of recovery. This study would indicate that versican plays a role in modulating hepatic fibrogenesis. It was also recently shown that proteolysis of versican by ADAMTS-5 constitutes a downstream mechanisms of the cerebral cavernous malformations (CCM) pathogenesis [105]. Exogenous expression of versican fragments increased sprouting and branching and were detected in close proximity of endothelial cells during CCM lesion growth. In a stark contrast, depletion of versican reduced CCM formations.

Versican has also been associated with the progression of tumor processes. In general, an increased expression of this hyalectan predicts a poor outcome for different types of cancer [106,107]. Versican influences cell migration and proliferation as well as angiogenesis and inflammation processes thus contributing to create a permissive ECM for invasion and metastasis of tumor cells. However, cleavage by ADAMTSs can modify the tumor-promoting functions associated with versican as has been shown by Hope et al. in myeloma [108]. In this type of tumor, macrophages produce versican V1 to induce a tolerogenic response mediated by the production of the type II cytokines interleukin-4 (IL-4), IL-5 and IL-10 due to the interaction of the hyalectan with antigen-presenting cells. On the other hand, production of ADAMTS-1 by mesenchymal stroma cells changes this scenario since the metalloprotease cleaves versican at the E441-A442 bond. The generated NH_2_-terminal fragment activates T-cells and induces an immunogenic response by production of the interleukins IL-6, IL-1β, IL-12p40 and CCL2. Consecuently, the production of versican or the generation of its bioactive 70-kDa versican V1 could drastically modify the outcome of the tumor [109]. Therapeutic administration of this bioactive fragment, now called versikine, could open new strategies in the immunotherapy of myeloma [108]. Moreover, immunoprotective function elicited by versikine has also been shown in solid tumors as colorectal cancer [106]. A growing body of evidence suggests that versican and its proteolytic products are active modulators of the tumor microenvironment promoting pro or antitumor effects by different mechanisms [81,110,111]. Also, participation of particular ADAMTSs in these processes and in different tumors has been shown or proposed to occur [112,113].

Versican can be cleaved by ADAMTSs at sites other than the E441-A442 of the V1 isoform. These cleavages would also generate bioactive fragments which could participate in other biological functions. For instance, the versican V0 fragment containing the DPEAAE-COOH neoepitope has also been associated to hepatic fibrosis [20]. Versican V0 and also Versican V2 can be cleaved at the E405-Q406 bond to generate a fragment named glial hyaluronan-binding protein (GHAP), identified in the human brain [114,115]. These and other fragments generated by versican cleavage, which are called versican-matrikines, would perform important biological functions that remain to be elucitated. Overall, versican or fragments generated by its proteolysis influence cell behaviour in a context-dependent manner [116], and would endorse the accuracy of the name versican for the versatility of functions associated with this hyalectan [117].

### 4.3. Cleavage of Brevican by ADAMTSs

Brevican or brain-enriched hyaluronan binding (BEHAB) is expressed in the CNS, and produced by glial cells and neurons [118,119]. This hyalectan is involved in physiological processes affecting brain plasticity, and it is located in perineuronal nets and in the axon initial segment [120]. In this sense, it has been found that, while hyaluronan is highly enriched in amyloid plaques, aggrecan-based perineuronal nets or brevican-based perisynaptic axonal coats show no apparent signs of alteration in Alzheimer´s disease [121]. Moreover, loss of brevican is related to loss of synapsis. These effects would indicate a protective effect of PGs in neuron degeneration. To perform these functions, brevican interacts in the ECM with components such as tenascin-R [122], neurofascin 186 [123] or Bral2 [124], thus contributing to organize extracellular space in the brain. The importance of brevican in CNS function is also evident when it has been shown that its expression increases following CNS injury, indicating its participation in the regeneration process [125]. Thus, low expression level of this hyalectan is detected in rat spinal cord in normal conditions. However, brevican can be moderately detected by immunolabeling 24 h after an injury is induced, continues to increase two weeks later, and remains elevated two months postinjury. Other experimental CNS injury models have also shown an increase not only of brevican but also of the other hyalectans [126,127]. Brevican would form part of an orchestrated molecular response for the regeneration process following traumatic injury [128]. In this regard, growth of axons is regulated by a balance of permissive and inhibitory factors to guarantee a successful regeneration. Brevican and other hyalectans would contribute to cause an inhibitory response during axon growth, and further removal of PGs would help to facilitate restoration of sensory function [126]. Moreover, factors that promote axon growth, as is the case of decorin [129], can also suppress deposition of brevican and other PGs to avoid their inhibitory effect.

**Figure 5:**
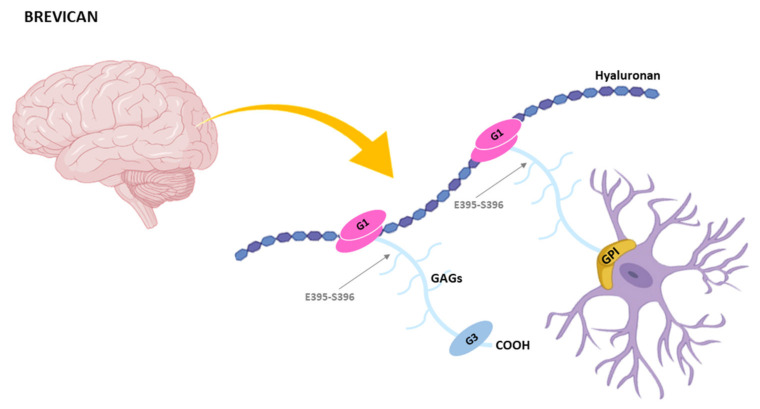
Representation of brevican structures. Brevican is expressed in brain and a GPI-anchored isoform lacking G3 domain has been identified. GAGs, glycosaminoglycans. Arrows indicate the main cleavage site by ADAMTS-4. Other cleavage sites are indicated in the text.

Brevican is the shortest member of the hyalectans and this structural feature is what its name refers to [118]. Like the rest of hyalectans, brevican contains two globular domains G1 and G3 at the NH_2_-terminal and COOH-terminal ends respectively. Between these domains, the central region comprises 1–5 potential glycosaminoglycan attachment sites [119] (Figure 5). It is also noteworthy that brevican is the only hylectan that exists in an alternatively spliced isoform containing a GPI anchor attachment site at the COOH-terminal end, while it lacks the G3 globular domain [130].

Proteolytic cleavage of brevican by different metalloproteases has been extensively reported [14,44,92]. In 2000, Nakamura et al. [131] showed that different members of the MMPs and ADAMTSs family could cleave brevican but at different sites. Thus, MMPs preferentially attacked the A360-F361 bond, while ADAMTS-4 cleaved the E395-S396 bond within the central region of brevican. In addition, other bonds can also be hydrolyzed by these enzymes. In that work it is also pointed out that brevican cleavage showed similarities with the aggrecan cleavage by these metalloproteases and it is suggested that both MMPs and ADAMTSs are responsible for brevican degradation in physiological and pathological conditions. For instance, brevican cleavage by ADAMTSs has been associated to progression of the gliomas [132], and an increased expression at both RNA and protein levels of ADAMTS-4 and ADAMTS-5 has been detected in glioblastoma, the most aggressive form of glioma [133,134]. Other findings supporting the role of brevican cleavage by ADAMTSs in this type of tumor is that the highly invasive rat CNS-1 glioma cell line produces and cleaves brevican when grown as an intracranial graft [135]. Moreover, generation by mutagenesis of brevican that cannot be cleaved by these ADAMTSs is unable to promote CNS-1 cells invasion in vitro and in vivo [136]. Also, the non-invasive rat 9 L glioma cells become invasive when producing a recombinant brevican fragment corresponding to the NH_2_-terminal end [137]. Association of brevican with glioma progression has opened the possibility that this hyalectan can be taken into consideration as a target for immunotherapy [138].

Fragments generated by the cleavage of brevican by ADAMTSs are also involved in other functions like remodeling of the dentate outer molecular layer after an exocitotoxic lesion [139]. Moreover, a recent study has shown that fragments generated by brevican cleavage in cerebrospinal fluid significantly decreased in patients suffering traumatic brain injury as compared with idiopathic normal pressure hydrocephalus patients [140]. Attending to the obtained results, it is proposed that the NH_2_-terminal and the COOH-terminal parts of brevican are differently regulated as a consequence of activities mediated by ADAMTSs, which could be employed as outcome markers following traumatic brain injury. In relation to Alzheimer´s disease and other dementias, it has been proposed that differential levels of brevican and the fragments generated after ADAMTS-4 proteolysis could be employed to distinguish among different types of dementias [141]. Moreover, ADAMTS-4 participates in functional recovery of spinal cord injury through the cleavage of different proteoglycans, including brevican [142]. Recently, it was also shown that neuromodulation of dopamine receptor induces proteolysis of brevican by ADAMTS-4 and ADAMTS-5, which could have repercussions for addictions and other diseases of the brain [143]. It is also important to note that not only ADAMTS-4 and ADAMTS-5 could cleave brevican. In this regard, mice lacking aggrecanase-1 or aggrecanase-2 show “brevicanase” activity [144]. Moreover presence of ADAMTS-1, ADAMTS-9 and ADAMTS-15 is detected in mice lacking ADAMTS-4 during spinal cord injury. Consequentely, this subset of ADAMTSs could also participate in the cleavage of brevican. Taking together, studies about the shortest hyalectan highlight the influence of its proteolysis by ADAMTSs in brain functions. But these studies also point out that other roles of brevican in the brain remain to be identified.

### 4.4. Cleavage of Neurocan by ADAMTSs

Neurocan is predominantly expressed in the central nervous system [145,146], and in a development-dependent manner. Thus, neurocan is detected at low level in the adult mouse brain in physiological conditions. However, it has been detected at embryonic day 12 in rats; and its expression increases in late embryogenesis stages but decreases in one month after birth [147]. Nevertheless, other hyalectans such as brevican or aggrecan are scarcely detected in the rat neonatal brain [148]. Regarding the specific role of neurocan in brain function, it was shown that it plays an important role in the formation of perineuronal nets in the auditory brainstem during postnatal development in mouse [149]. Moreover, neurocan inhibits specific signaling pathways, thus contributing to postnatal remodeling of interneuron axons [150], or to hamper the spine remodeling mediated by semaphorin 3F in cortical neuronal cultures [151]. These examples illustrate the relevance of the interactions of neurocan with other components of the brain ECM. In this sense, it is noteworthy that a neurocan-deficient mouse does not show any apparent functional deficits [152]. Nonetheless, mice lacking neurocan, brevican, tenascin-C and tenascin-D show alterations in both excitatory and inhibitory synaptic responses [153]. Both tenascins are interacting partners of neurocan within the brain ECM [154]. In addition to its participation in normal physiological processes, the neurocan level considerably increases following brain damage [155], and it is produced by reactive astrocytes in chronic glial scar [156]. This increment would indicate the participation of neurocan in the regeneration process since this hayalectan hampers neurite outgrowth. Moreover, neurocan can be detected in both soluble and insoluble forms two months post-injury [157]. Taken together, these findings strongly suggest that neurocan could form part of a mechanism that regulates regeneration of the damaged tissue. Interaction of neurocan with cell adhesion molecules such as Ng-CAM or N-CAM [158,159], could be involved in this process [160]. Effects of neurocan on cell adhesion can also be inferred from the fact that its exogenous expression in adherent neuroblatoma cells induce formation of spheres, thus increasing malignancy in vivo [161].

**Figure 6:**
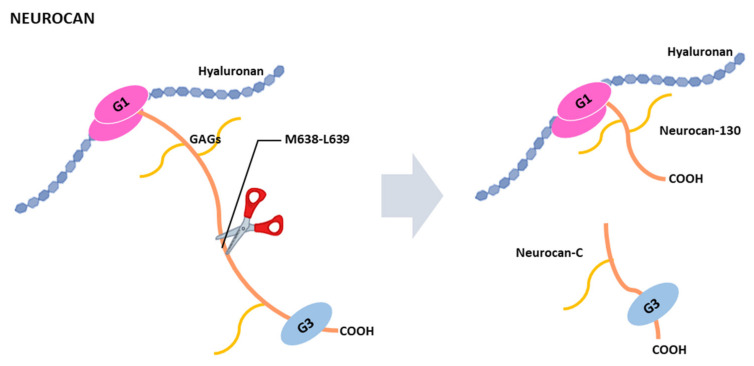
Schematic representation of neurocan proteolysis. Neurocan is cleaved at M638-L639 to generate two fragments, neurocan 130 and neurocan-C, corresponding to the NH_2_-terminal and COOH-terminal ends of the original undigested neurocan. G1 and G3 indicate globular domains. Other cleavage sites are indicated in the text.

Like aggrecan and brevican, neurocan contains two globular domains, G1 and G3, separated by a central region containing six potential sites for glycosaminoglycans attachment [146] (Figure 6). This central region undergoes proteolytic processing at the M638-L639 bond to release two fragments, called neurocan-130 and neurocan-C [154,155]. This processing was detected in culture astrocytes and it is not affected by the addition of serine, cysteine or metalloprotease inhibitors, suggesting that the processing occurs intracellularly [155]. Besides this processing, other cleavages take place within the neurocan core protein since different fragments are produced in cell lines or tissue samples [159]. For instance, a 90 kDa fragment was detected in rat brain neurocan [154,162], or a 45 kDa fragment was identified in one case from adult brain [146]. Some proteolytic enzymes have been shown to cleave neurocan such as the serine protease plasmin [163], or the metalloproteases MMP-2, ADAMTS-4, ADAMTS-5 and ADAMTS-12. MMP-2 cleavage corresponds to that producing the neurocan-130 and neurocan-C fragments [164]. ADAMTS-4 can also degrade neurocan, thus reversing the inhibitory effect induced by the hyalectan on neurite outgrowth [142]. Our previous work also point to that ADAMTS-5 and ADAMTS-12 can cleave neurocan in vitro [160]. As indicated for ADAMTS-4, ADAMTS-12 activity could contribute to the clearance of neurocan to re-establish neurite outgrowth following a brain injury. However, neurocan cleavage by ADAMTS-12 generates a 50 kDa fragment and different experimental approaches indicate that this fragment affect adhesive properties of the H4 human neuroglioma cell line. Even more, gene coding for ADAMTS-12 is associated with schizophrenia [165], as occurs with neurocan [166]. An inefficient cleavage of neurocan by ADAMTS-12 could contribute to the risk of mental disorders [160]. Despite the significant advances in the functions performed by neurocan in recent years, consequences of its clearance by ADAMTSs or in the generation of bioactive fragments remain to be explored. In this regard, characterization of “neurocanase” activities by ADAMTSs could help to better understand the role performed by the multiple interactions of neurocan within the brain ECM.

## 5. Concluding Remarks

In this article we tried to summarize the consequences of the cleavage of hyalectans by ADAMTSs metalloproteases. As can be appreciated, these consequences are many, take place in different tissues and at different levels. Even more, opposite effects can be exerted depending on the hyalectans and the way they are processed. An example is the ADAMTS proteolytic activity on brevican that promotes protumor effects in glioma; but cleavage of versican by ADAMTSs produces versikine, which displays antitumor activity in myeloma. This is indicative that some important advances on the consequences of hyalectans cleavage by ADAMTSs have been achieved. However, mechanisms of hyalectans accumulation, clearance or proteolytic processing are far from clarified. It is only necessary to take into account that twenty years have passed since aggrecanases were considered therapeutic targets in arthritic diseases. Initial strategies based on the use of small molecules containing a chemical group to block the active site of these enzymes lacked success due to unspecificity and bioavailability troubles. In current times, a plethora of studies on aggrecanolysis mediated by ADAMTS opens the possibility to develop new strategies based on the specific blocking of regions within the ancillary domain which are involved in substrate recognition. For instance, employment of humanized antibodies against these regions within ADAMTS-5 molecular architecture is a current challenge that could offer satisfactory clinical results. Biology of ADAMTSs is more complex than initially thought. Still, investigations on these metalloproteases and some of their preferred substrates, the hyalectans, should shed light about the physiology not only of the articular cartilage, but also related to the cardiovascular or central nervous system. In particular, the possibility that different bioactive fragments can be generated from the cleavage at different positions of the four hyalectans should be explored in depth. Furthermore, these investigations should serve to offer more adequate therapeutic options to avoid the unwanted effects of an inappropriate hyalectanase activity.
